# Discovering Engagement Personas in a Digital Diabetes Prevention Program

**DOI:** 10.3390/bs12060159

**Published:** 2022-05-24

**Authors:** Jonathan H. Hori, Elizabeth X. Sia, Kimberly G. Lockwood, Lisa A. Auster-Gussman, Sharon Rapoport, OraLee H. Branch, Sarah A. Graham

**Affiliations:** Lark Health, 2570 W El Camino Real, Mountain View, CA 94040, USA; jonathan.hori@lark.com (J.H.H.); liz.sia@lark.com (E.X.S.); kimberly.lockwood@lark.com (K.G.L.); lisa.austergussman@lark.com (L.A.A.-G.); sharon@lark.com (S.R.); oralee.branch@lark.com (O.H.B.)

**Keywords:** digital health, mHealth, behavior change, type 2 diabetes, clustering

## Abstract

Digital health technologies are shaping the future of preventive health care. We present a quantitative approach for discovering and characterizing engagement personas: longitudinal engagement patterns in a fully digital diabetes prevention program. We used a two-step approach to discovering engagement personas among *n* = 1613 users: (1) A univariate clustering method using two unsupervised k-means clustering algorithms on app- and program-feature use separately and (2) A bivariate clustering method that involved comparing cluster labels for each member across app- and program-feature univariate clusters. The univariate analyses revealed five app-feature clusters and four program-feature clusters. The bivariate analysis revealed five unique combinations of these clusters, called engagement personas, which represented 76% of users. These engagement personas differed in both member demographics and weight loss. Exploring engagement personas is beneficial to inform strategies for personalizing the program experience and optimizing engagement in a variety of digital health interventions.

## 1. Introduction

Digital technologies are transforming health care and making preventive care broadly accessible to meet the needs of a growing population [1]. The COVID-19 pandemic further spurred the need for and emergence of digital care options [2]. One example of a preventive health care program that is increasingly being offered via digital platforms is the National Diabetes Prevention Program (DPP), a traditionally in-person program focused on preventing or delaying the onset of type 2 diabetes through lifestyle changes [3,4].

Primary prevention of diabetes and its complications is an important public health priority. More than 96 million Americans have prediabetes [5], with an 11% annual conversion rate to type 2 diabetes reported in the follow-up DPP Outcomes Study [6]. The annual health care costs for an individual with diagnosed type 2 diabetes are more than double those for an individual without diabetes [7]. The National DPP can cut the risk of progressing to type 2 diabetes in half by educating participants on healthy lifestyle behaviors and facilitating weight loss [3]. Participants who actively engage with the educational content of the DPP and adopt self-management behaviors, such as frequent weighing and tracking of diet and physical activity, can achieve clinically meaningful weight loss [8].

Digital DPPs have the potential to reach a large portion of the population. In 2021, 85% of individuals in the US reported owning a smartphone [9], which is a primary means of accessing digital programs. The remote intervention delivery of a digital DPP can overcome commonly cited barriers to engagement in face-to-face programs such as accessibility, transportation, and scheduling [10]. Digital programs are further able to provide continuous monitoring and support and timely communication of health information, which are critical components of successful behavior change programs [11]. However, there are also barriers to sustained engagement in digital programs, such as barriers to usability and lack of accountability to a human coach, and digital programs have reported high attrition rates [12]. To promote engagement sufficient for achieving clinically meaningful outcomes, it is important to tailor a personalized program experience that can meet the needs of the diversity of members who choose a digital DPP [13]. This personalization requires a deeper understanding of how different users engage with digital health programs and application-specific components. Prior investigations of engagement in mobile- and web-delivered interventions for adults with type 2 diabetes considered discrete engagement variables in isolation (e.g., average number of website logins) [14]. Although these types of reports lend important information about the amount of engagement in a program, they do not describe the unique ways that individual users interact with program content and application features. A more holistic description of the user “engagement persona” would provide a deeper understanding of engagement patterns that reflect actionable ways to personalize the program experience for different types of users.

User personas are commonly used in user experience research to highlight salient characteristics that represent the characteristics and/or needs of a larger group of users or research participants. User personas are traditionally fictitious, and they are defined based on the expectations of user experience researchers or interview responses from a few archetypal members [15]. User personas can be developed using a wide variety of factors, such as demographics, behaviors, values, goals, or motivations. There is limited research exploring user personas in digital health engagement, with most existing work focused on member retention in the first days of digital health interventions (e.g., [16]). Notably, there is little research on engagement patterns with digital health technologies over time. Assessing engagement over time is particularly important, as sustaining engagement remains the most difficult part of intervention implementation. The few studies available suggest that patterns of engagement over time are related to health outcomes such as cardiovascular symptoms, weight loss, and anxiety or depression [17,18,19].

To further understand user engagement in digital health over time, there is a need for additional research on user personas and their utility. Specifically, new techniques are needed for discovering distinctive, longitudinal engagement patterns or engagement personas. We propose that data-driven approaches using longitudinal engagement data can be the most informative for discovering engagement personas. A data-driven approach can identify salient features of members based upon real-time behavior and actions that do not necessarily fit qualitative predeterminants. The detailed information from each member’s time series of interactions with a program enables the discovery of engagement personas using quantitative techniques that minimize human bias [20]. Engagement personas reflect an affinity for program components and highlight actionable ways to further personalize the program experience for each member and encourage meaningful and sustained engagement. Given that the National DPP is a highly used behavior change program that has recently expanded its digital offerings, a digital DPP provides an ideal opportunity to examine digital health engagement personas. The discovery and application of engagement personas represents a key area of research that can optimize the delivery of digital DPPs as the demand for these programs rapidly grows.

The goal of the present study was to quantitatively identify engagement personas based on distinctive, longitudinal engagement patterns in a fully digital DPP that is powered by conversational artificial intelligence. We employed a hypothesis-generating, inductive approach; thus, we did not have an a priori hypothesis as to the number of engagement personas we would discover. However, we did expect to observe significant differences in clinical outcomes between engagement personas that differed in their amount of engagement. For example, we hypothesized that members belonging to engagement personas that exhibited higher overall engagement would lose a greater amount of weight than members of engagement personas characterized by lower overall engagement. Finally, to validate the discovered engagement personas and investigate this hypothesis, we compared characteristics and weight loss between engagement personas and tested the persistence of the engagement personas both with an alternative algorithm and on an independent set of data.

## 2. Materials and Methods

### 2.1. Program Overview

This study used data from a fully digital program called the Lark DPP, which has thousands of members participating monthly. This DPP has full recognition from the Centers for Disease Control and Prevention (CDC, Atlanta, GA, USA) (organization #4358176), which is the highest level of DPP certification based on approved curriculum, enrollment, engagement, and clinical efficacy outcomes. The educational content follows the PreventT2 curriculum [5] through educational lessons centered on losing weight through lifestyle changes. Members engage with mobile app features, educational content, and coaching that facilitate positive behavior changes such as weight loss, physical activity, a healthy diet, and managing stress and sleep.

Lark’s coaching of these healthy behaviors is unique among the digital DPPs recognized by the CDC because it is powered by conversational artificial intelligence. Human experts and coaches designed the coaching conversations using evidence-based strategies including cognitive behavioral therapy and positive psychology. An artificial intelligence coach is uniquely positioned to provide personalized coaching since it can retain a perfect memory of all interactions with a member and refine coaching, feedback, and mechanisms of delivery as it learns the patterns and preferences of each member. Thus, an artificial intelligence platform is ideally suited to facilitate the discovery of engagement personas because it gathers and stores detailed information about individual patterns of use.

The coaching in this digital DPP imitates a human coaching experience and provides (1) An educational curriculum on behavior change related to diabetes prevention, (2) Feedback and insights on goals and health behaviors, and (3) Daily and weekly summaries of member progress (e.g., dietary changes, weight loss) so that users can track their progress over time. The artificial intelligence coach engages members in synchronous, text-based coaching conversations, so users can have a coaching exchange any time they open the app.

### 2.2. Participants and Recruitment

Members enrolled in the digital DPP as a covered service under their health insurance plans. All members agreed to the app’s privacy policy at registration, which included permission to use their de-identified data for research. This study received exemption status from the Advarra Institutional Review Board (Columbia, MD, USA, #Pro00047181) for retrospective analyses of de-identified data. Members had to meet CDC requirements for prediabetes: (1) Aged 18 years or older, (2) No previous diagnosis of type 1 or 2 diabetes, and (3) Initial body mass index ≥ 25 kg/m^2^. Members also had to meet one of the following: (1) Blood test result (within the past year) of fasting plasma glucose between 100 and 125 mg/dL, two-hour plasma glucose after 75 g glucose load between 140 and 199 mg/dL, or hemoglobin A1c of 5.7–6.4% or (2) Risk score from CDC’s online risk assessment indicating high risk for type 2 diabetes [21]. CDC requires that at least 35% of participating members for a given organization report a blood test result within the past year indicating prediabetes. The remaining members may qualify based solely on the CDC online risk assessment.

We used data from members who enrolled in this digital DPP between 1 August 2019 and 1 October 2019. Using this time frame ensured that all members included in the analyses experienced the same version of the application and program features. A total of 2938 members enrolled during this time frame and downloaded the mobile application to their smartphones. For a member to be included in the analyses they also had to show extended engagement, defined as having at least one bidirectional conversation with the artificial intelligence coach after their first 90 days in the program. A total of 1613 members met this criterion for extended engagement. We required extended engagement for a couple of reasons. First, although this period of engagement was longer than other studies adopted (e.g., [16]), it enabled us to focus our results on longitudinal engagement patterns and not simply cluster by variations in member drop-off time. Second, the National DPP has shown that the highest attrition rates occur immediately after the first week and between weeks 16 and 19 [22]. We similarly found that the percentage of members who disengaged or uninstalled the app in the next 30 days (i.e., churn) reached a minimum at 90 days. Thus, 90 days provided a time frame well after initial drop-off and long enough to observe longitudinal patterns of use.

### 2.3. Measures

#### 2.3.1. Demographics

Members entered their demographic data and additional characteristics in-app, including their age, gender, ethnicity, and height. All members included in the present analyses received a digitally connected scale upon enrollment that automatically uploaded their body weight to the app. We assessed starting body mass index (kg/m^2^) at program initiation using each member’s height and starting weight.

#### 2.3.2. Weight Loss

The primary clinical outcome was percent weight loss at four months in the program. We selected this outcome because percent weight loss is the primary clinical outcome in CDC-recognized DPPs. Achieving ≥5% weight loss during the program is associated with a 58% reduction in the risk of converting to type 2 diabetes [3]. Assessing weight loss at the interim time point of four months provided insight into the potential program impact after the minimum 90-day engagement window described above.

#### 2.3.3. Engagement Metrics

We focused on four primary metrics to assess engagement with the program: the number of coaching exchanges with the artificial intelligence coach, meals logged, weigh-ins, and educational lesson check-ins. We selected these four engagement variables to prioritize features related to weight loss [23], which is the primary objective of the DPP. Coaching exchanges could occur any time the member opened the app and provided the core structure for coaching interactions, encouragement, and feedback delivery.

Natural language processing powers the meal logging features in-app. Members enter meals via free text using straightforward, everyday language (e.g., I ate half a turkey sandwich). The app sends meal logging reminders to members outside of coaching exchanges to encourage members to log meals.

Members use their connected digital scales to complete weigh-ins during the program. Members can weigh themselves as often as they wish but are encouraged to complete at least one weigh-in per week.

Lesson check-ins refer to the completion of portions of educational lessons within the app that correspond to the National DPP PreventT2 curriculum [21]. The digital DPP used in this study spreads the educational content of a single lesson over seven check-ins (one per day) to deliver information to members in digestible amounts.

#### 2.3.4. Creation of Engagement Time Series Variables

We created four time series variables from the above-detailed engagement metrics: (1) The number of days per week with coaching exchanges, (2) The number of days per week with meals logged, (3) The number of days per week with a weigh-in, and (4) Whether a member checked in each day (0 = no; 1 = yes) with the educational lessons of the program. Time series 1–3 were “app features” and captured the major ways that a member could engage with the app. These time series were each 13 elements long per member, corresponding to the weeks (1–13) in the first 90 days of the DPP. Since there was no limit to the number of coaching exchanges, meals logged, or weigh-ins a member could have, we aggregated these app features weekly to capture high-level, cross-app engagement while reducing sensitivity to fluctuations in daily engagement. We further concatenated these app-feature time series into one 39-element series (i.e., 13 × 3) to simplify the model and focus our clustering on the general shape of members’ app-wide engagement without emphasizing any single feature a priori. Time series 4 was a “program feature” reflecting the most important data used to measure a member’s progress through the educational lessons of the DPP. The app only allows members to complete one check-in per day, so this time series was 90 elements long to capture higher-frequency, daily changes in program engagement.

### 2.4. Statistical Analyses

#### 2.4.1. Unsupervised Discovery of Engagement Personas

We employed a hypothesis-generating, bottom-up approach to discovering engagement personas; thus, we did not have an a priori hypothesis as to the number of engagement personas we would discover. We used a two-step approach to discovering engagement personas. The first step was a univariate clustering method using an unsupervised k-means clustering algorithm with a Euclidean distance metric. We conducted two separate univariate clustering analyses, one for engagement with app features and one for engagement with program features. The second step involved cross-referencing the app- and program-feature univariate clusters to reduce noise in the final engagement personas and identify members who were strongly related across both app and program features. Each step is detailed below.

#### 2.4.2. Univariate Cluster Analyses

We independently clustered the data for each time series: first for the concatenated weekly app-feature time series and second for the daily program-feature time series. We log-scaled the app features after observing improved cluster fit for the log-scaled vs. raw data. This was likely because the log-scaled data captured the percent difference between members’ frequencies of app actions rather than the absolute distance. For example, members engaging more frequently in an app behavior (e.g., 6 vs. 7 coaching exchanges/week) were likely more similar than members engaging less frequently in that same behavior (e.g., 1 vs. 2 days/week).

We chose independent clustering steps for app features and program features to maximize the flexibility of engagement patterns represented by our clusters and enable each group of features to retain a unique time scale of observation (i.e., weekly vs. daily). The general program feature also enabled this approach to be program agnostic. App features represent items that the Lark program has control over within the app: the amount of coaching, weigh-in reminders, and meal logging reminders. For the National DPP, the program material is defined by the PreventT2 curriculum. If a different program used this method for discovering engagement personas, the app-feature clustering could be cross-referenced to the educational content of a different program, as described below under “Bivariate Clustering Method”.

Without an a priori expectation for an optimal number of clusters, we evaluated the resulting clusters at several candidate choices of k and selected the final value when the clusters were granular enough to capture meaningful differences in engagement patterns while remaining representative enough to avoid overfitting. For each univariate clustering step, we independently determined the optimal number of clusters, k, using silhouette coefficients and post hoc analyses. The silhouette coefficient ranged from 1 (best match to its own cluster) to −1 (worst match), with 0 indicating near-overlapping clusters. When determining the final k, we used the average silhouette coefficient of each cluster and selected a k that resulted in the highest coefficient per cluster.

#### 2.4.3. Bivariate Clustering Method to Determine Engagement Personas

The bivariate clustering method involved comparing the cluster label for each member across both univariate clustering steps. We constructed a resulting contingency table of cluster label counts per each label pair (Table 1). We identified engagement personas based on cells in Table 1 that contained some predefined proportion of each label’s marginal total members. We mathematically denoted #(*a*, *b*) as the number of members with app-feature cluster label “a” and program-feature cluster label “b”. Then, for each label pair (*x*, *y*), we identified an engagement persona if:

$$\frac{\#\left(x,y\right)}{\sum_{i=1}^{k_{app}}\#\left(i,y\right)}\geq T\;\mathrm{or}\;\frac{\#\left(x,y\right)}{\sum_{j=1}^{k_{program}}\#\left(x,j\right)}\geq T$$

where *k_app_* and *k_program_* are the numbers of clusters per method, and *T* is the minimum required percentage of the marginal total number of members. We determined the final cross-referencing threshold by evaluating the average silhouette coefficient among all members belonging to clusters that resulted in an engagement persona at each cross-referencing threshold and further selecting a threshold that captured a large percentage of members. We performed Mann–Whitney U tests to compare the average silhouette coefficients of members with versus without an identified engagement persona for each of the univariate clusters.

#### 2.4.4. Demographics and Characteristics of Engagement Personas

We used Tukey post hoc tests to compare participant demographics and characteristics (age, gender, race, ethnicity, and body mass index), aggregate engagement counts (coaching exchanges, meals logged, weigh-ins, lesson check-ins), and clinical outcome (weight loss at 4 months) between the engagement personas. We also compared engagement personas on membership in the digital DPP’s concurrent Facebook group to better understand if certain engagement personas were more inclined to seek social support from peers.

#### 2.4.5. Statistical Validation of Engagement Personas

We statistically validated the engagement personas to ensure that the distinctive longitudinal engagement patterns, which represent the unique ways that members engaged with the digital DPP, were both representative and consistently discoverable. This validation approach consisted of two steps: (1) Using an alternative clustering algorithm called hierarchical agglomerative clustering with Ward’s method and (2) Applying the same k-means clustering method on an independent test sample of 1071 DPP members from the same program using a different enrollment window of 1 February 2020 to 30 June 2020.

## 3. Results

### 3.1. Univariate Clusters

**Figure 1:**
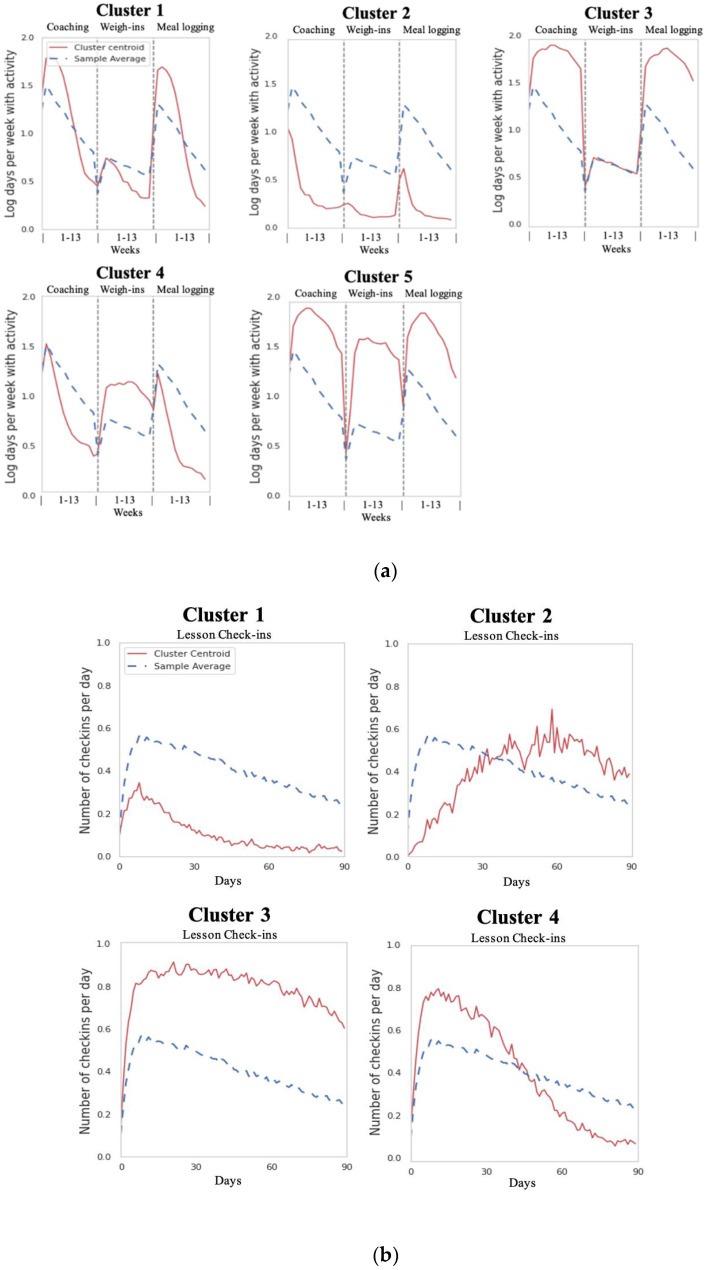
(**a**) Five concatenated univariate app-feature clusters showing log-scaled days per week (y-axis) with activity for coaching exchanges, weigh-ins, and meal logging over 13 weeks in the digital DPP (x-axis). Key (top left panel): Cluster centroids shown in red lines and sample average in blue, dotted lines. (**b**) Four program-feature clusters showing daily check-ins (y-axis) with educational lessons over the 90 days (x-axis) in the digital DPP. Key (top left panel): Cluster centroids shown in red lines and sample average in blue, dotted lines.

As described above, when determining the final k, we used the average silhouette coefficient of each cluster and selected a k that resulted in the highest coefficient per cluster. Using this criterion, the univariate analysis revealed five app-feature clusters (Figure 1a) and four program-feature clusters (Figure 1b). A cluster centroid above the sample average indicated that members in that cluster engaged with a particular feature more than the rest of the sample and vice versa. Changes in the level and slope of the centroids provided information about when cluster members tended to increase or decrease their engagement with a feature.

The app-feature clusters revealed stratification across both the level of engagement and the type of engagement with app features. We discovered clusters of members who had high and low engagement relative to the sample average across all app features and members whose engagement differed depending on the app feature (Figure 1a). Weigh-ins were a distinguishing feature among app-feature clusters. For example, app-feature clusters 3 and 5 contained members with higher-than-average coaching exchanges and meal logging (centroid lines above sample means for clusters 3 and 5 in Figure 1a). However, members in cluster 3 only had an average frequency of weigh-ins (centroid line on top of the sample mean in Figure 1a) compared to those in cluster 5 who exhibited a higher-than-average frequency of weigh-ins. Members in cluster 4 also had a higher-than-average frequency of weigh-ins, despite having lower-than-average engagement across the other app features (centroid line below the sample mean for coaching exchanges and meal logging in Figure 1a).

The program-feature clustering revealed clear stratification of engagement with the educational lessons of the DPP. There were four program-feature clusters: two clusters emerged representing consistently low and high engagement with lesson check-ins over 90 days relative to the sample average (see Figure 1b centroid line for clusters 1 and 3), and two additional clusters represented members whose engagement with check-ins increased and decreased steadily over 90 days (see Figure 1b centroid line for clusters 2 and 4).

### 3.2. Bivariate Clusters: Identification of Engagement Personas

**Figure 2:**
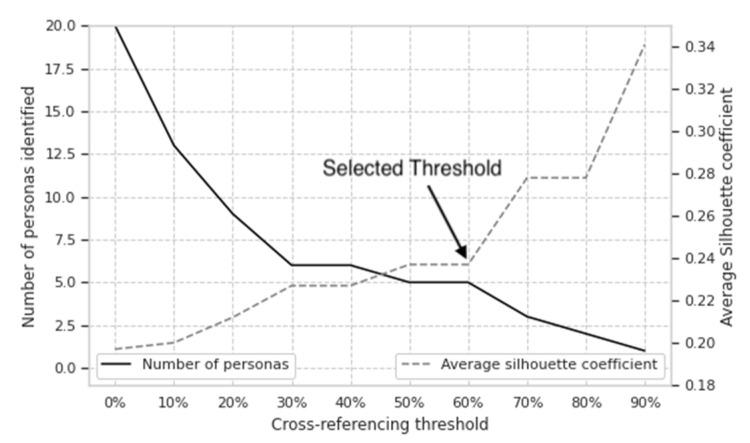
The number of engagement personas (solid, black line; left y-axis) for each cross-referencing threshold (x-axis) and the corresponding average silhouette coefficient (gray, dashed line; right y-axis). We selected 60% based on obtaining the highest silhouette coefficient that captured a large percentage (76%) of members. Lowering to 40%, for example, only increased the percentage of members to 80% but would have added an additional engagement persona with a small *n* that overfitted the data. In contrast, raising it to 70% would have dropped the percentage of captured members to 52%.

For the bivariate clustering method, we used a cross-referencing threshold of 60% to combine the univariate clusters and identify engagement personas. We selected 60% after evaluating the average silhouette coefficient of members belonging to clusters that resulted in an engagement persona at many different cross-referencing thresholds (Figure 2). We selected the threshold with the highest silhouette coefficient that also captured a large percentage of members. Although a cross-referencing threshold of 30–40% would have captured more members and had a similar silhouette coefficient (Figure 2), the increase in members captured was small, and the added engagement persona would have likely led to overfitting of the data. There was no difference in the magnitude of the silhouette coefficient or percentage of members between 50% and 60% (Figure 2), so we selected the higher of the two thresholds.

To apply the bivariate clustering method with the 60% threshold, we constructed a resulting contingency table of cluster label counts per each label pair (Table 1). We discovered five bivariate clusters or engagement personas that represented 1219 members—76% of the members who had extended engagement with the digital DPP. The remaining 24% of members did not fit within these five distinctive patterns of engagement. The bivariate clustering step served to eliminate cluster label pairs that did not capture many members, regardless of how well these members may have fit into their respective app-feature or program-feature univariate clusters.

The qualitative assessments of each univariate cluster centroid against the sample-wide time series averages (Figure 1a,b) facilitated the description of each engagement persona. The descriptive names assigned to each engagement persona embodied the distinctive patterns of engagement with both app and program features combined. The resulting engagement personas were: (1) Casual Members: Those who had lower app and program engagement than the sample average, (2) Mainstream Members: Those who had initially higher app and program engagement than the sample average but whose engagement with both features dropped substantially over 90 days, (3) Learners: Those who had higher program engagement than the sample average and higher engagement with coaching exchanges and meal logging but who had a weigh-in frequency consistent with the average, (4) Data-Driven Members: Those who weighed in more than average but had lower program engagement than the sample average and average engagement with coaching exchanges and meal logging, and (5) Enthusiasts: Those who had consistently higher app and program engagement than the sample average.

When cross-referencing the univariate clusters for the bivariate clustering step, we observed that members belonging to an app-feature cluster with high engagement also tended to be in a program-feature cluster with high engagement. For a member who had their engagement patterns captured by an engagement persona, knowing the member’s app-feature cluster label uniquely determined the member’s program-feature cluster label. For example, if a member fell into app-feature cluster 1, the member had a program-feature cluster label 4, identifying them as a Mainstream Member.

Conversely, knowing a member’s program-feature cluster label only narrowed the set of possible app-feature clusters. For example, members with higher program engagement than the sample average (program-feature cluster 3) might be Learners (combined with app-feature cluster 3) or Enthusiasts (combined with app-feature cluster 5). Similar to the univariate clustering results, differences in the frequency of weigh-ins enabled us to distinguish the engagement personas. For example, a member with lower program engagement than the sample average had a lower frequency of coaching exchanges and meal logging, and vice versa, with only that member’s weigh-ins differentiating between each bivariate cluster and determining their final engagement persona.

**Figure 3:**
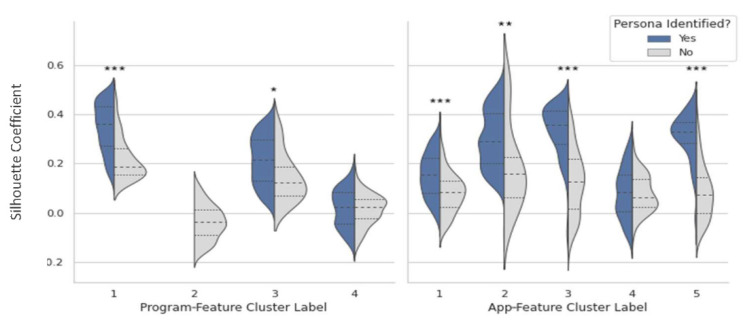
Violin plots of silhouette coefficient distributions with median (interquartile range) for the four program-feature clusters (**left**) and five app-feature clusters (**right**). The median silhouette coefficients (longer, dashed lines) for members with an identified engagement persona were higher than the medians for members without an identified engagement persona for all but program-feature cluster 4 and app-feature cluster 4. Mann–Whitney U tests between members with vs. without an identified engagement persona, * *p* ≤ 0.05; ** *p* ≤ 0.001; *** *p* ≤ 0.0001.

The bivariate clustering method identified engagement personas for members with the best fit in their respective univariate clusters. We compared the silhouette coefficients for the 1219 members with an identified engagement persona to those of the 394 members who did not belong to an engagement persona. Figure 3 displays the distribution of silhouette coefficients per univariate cluster label split by whether we were able to identify an engagement persona. Members without an identified engagement persona generally had the lowest silhouette coefficients, indicating a poor fit in their univariate clusters. Members in program-feature cluster 2 had particularly low silhouette coefficients compared to the other clusters, and this cluster did not have an associated engagement persona. Thus, the choice to have a higher number of univariate clusters, and capture greater nuances in engagement patterns, resulted in a cluster with poor fit and fewer members than the other clusters. The bivariate clustering method served to eliminate poorly fitting clusters and exclude them from an engagement persona. Results of the Mann–Whitney U tests comparing the average silhouette coefficients of members with versus without an identified engagement persona for each of the univariate clusters showed significant differences between all but two of the clusters (Figure 3).

**Figure 4:**
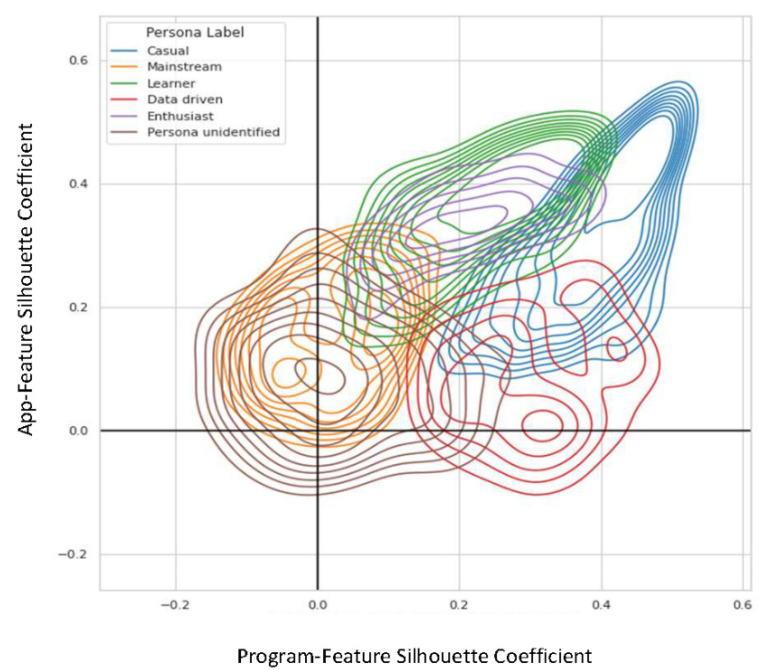
Kernel density estimation plot of the bivariate distribution of silhouette coefficients for each engagement persona. Members without an identified engagement persona (brown) had lower silhouette coefficients than those with an identified engagement persona.

For a display of the relationship between the goodness of fit for each cluster pair, Figure 4 shows a kernel density estimate plot of each member’s silhouette coefficients for both app- and program-feature clustering steps combined. Members with an identified engagement persona had higher silhouette coefficients for both univariate clusters (placing them in the upper right of the quadrants) compared to members without an identified engagement persona (lower left). Casual Members and Learners consisted of members who had high silhouette coefficients across both univariate clustering methods. Mainstream Members had relatively low silhouette coefficients compared to the other engagement personas.

### 3.3. Demographics and Characteristics of Engagement Personas

The engagement personas differed in aspects of demographics, characteristics, and clinical outcomes (Table 2). Enthusiasts and Learners tended to be older than the other engagement personas. Because percent weight loss is the primary clinical outcome in the DPP, weight-related differences across groups are important. Casual Members had the highest starting body mass index, and Enthusiasts had the lowest, though all engagement personas had a starting body mass index in the obese range. Enthusiasts showed significantly greater weight loss at four months compared to the other engagement personas, followed by Data-Driven Members and then Learners. The two engagement personas with engagement on the lower end of the spectrum, Mainstream Members and Casual Members, had the lowest percent weight loss. Casual Members also had the lowest representation in the program’s Facebook group.

### 3.4. Statistical Validation of Engagement Personas

We observed persistence in the engagement personas when using both the alternative hierarchical clustering approach on the initial *n* = 1613 training set of members and when using the same k-means approach on an independent test set of *n* = 1071 members (for details of the statistical validation analyses, see Appendix A).

## 4. Discussion

This study used a data-driven approach for discovering engagement personas among members of a CDC-recognized digital DPP. We identified five engagement personas that captured distinctive longitudinal features in member behavior, including the ways that members engaged with app and program features and how usage patterns evolved over time. We described these engagement personas as: (1) Casual Members, (2) Mainstream Members, (3) Learners, (4) Data-Driven Members, and (5) Enthusiasts. We also provided both clinical and statistical validation of these engagement personas. Notably, the engagement persona with the highest engagement (Enthusiasts) had the greatest weight loss compared to other engagement personas, followed by Data-Driven Members and Learners. Engagement personas with lower engagement (Casual Members) and engagement drop-off over time (Mainstream Members) had the least weight loss. These findings add to the growing body of literature examining digital health engagement and demonstrate that longitudinal patterns of engagement are linked with clinical outcomes, similar to the findings of research on other types of digital health programs [17,18].

In one recent study, the authors examined variability in cardiovascular monitor use and found four engagement patterns based on the amount of use over time: consistently high use, consistently moderate use, early high use with a rapid decline to low use, and early moderate use with a decline to low use [17]. There was an association between these engagement patterns and cardiovascular health outcomes; for instance, users with consistently high use tended to have more episodes of atrial fibrillation, and users with consistently moderate use tended to have more heart palpitations during the study. Other research went a step further and, like our work, attached descriptive engagement persona names (minimal users, activity trackers, dedicated all-around users, and all-around users with exceptional food logging) to usage patterns of self-monitoring technologies over time [18]. Users designated as “all-around users with exceptional food logging” showed the greatest weight loss of the four engagement personas. Our work also shares similarities with that of Chien et al., [19] who identified engagement patterns in an internet-based cognitive behavioral therapy program for improving symptoms of anxiety and depression and proposed tailoring interventions according to specific subtypes of engagement. A benefit of our method for discovering engagement personas is that we separated the app features and program features in the initial stage of clustering. This approach enables the application of our method to digital programs with different content (i.e., other than DPPs) but similar app features. To our knowledge, our study is the first to examine longitudinal engagement patterns derived from multiple types of engagement metrics in a digital DPP. Given that the resulting engagement personas were related to the primary clinical outcome of the program, weight loss, we speculate below how this information could be used to further tailor the program experience.

### 4.1. Utility of the Engagement Personas

The engagement personas captured key differences in member characteristics and clinical outcomes. Two of the discovered engagement personas represented higher and lower engagement, respectively, with all features of the digital DPP. Enthusiasts exhibited higher app- and program-feature engagement than the sample average. These members likely have high levels of intrinsic motivation [23] and are ideally suited for a digital DPP. Enthusiasts had both the lowest starting body mass index of the engagement personas and the greatest percent weight loss at 4 months. In contrast, Casual Members exhibited lower app- and program-feature engagement than the sample average. Casual Members had the highest starting body mass index, suggesting the highest risk for prediabetes [24], and the smallest magnitude of weight loss at 4 months. The differences in weight loss for these contrasting engagement personas support the literature that higher engagement may be necessary for achieving clinical outcomes [25,26]. Thus, strategies for increasing the engagement of Casual Members may be imperative. Casual Members may require more extensive outreach efforts and/or behavioral counseling than the other engagement personas to increase their engagement. Outreach efforts could serve to uncover psychological barriers (e.g., low intrinsic motivation) and then DPP lifestyle coaches could implement appropriate behavioral counseling techniques (e.g., motivational interviewing) [27]. Alternatively, programs could offer incentives targeted at encouraging the most beneficial behaviors for weight loss (e.g., self-weighing) [28].

We observed that Enthusiasts were significantly older than Casual Members. The National DPP has historically recruited large numbers of older adults (29.2% aged 55–64 years and 30.9% aged > 65 years) and demonstrated better retention and outcomes for older compared to younger members [22]. One reason younger adults may disengage early from a program (particularly a digital one) is a lack of connection with fellow program members [29]. Indeed, Casual Members had the lowest representation in the program’s Facebook group. Promoting social network participation may be one way to encourage Casual Members to increase their engagement [30]. Another more personalized possibility could be to pair new members who exhibit Casual Member patterns with a peer support person (e.g., an Enthusiast). Support from peers with similar lived experiences has been shown to be an effective means of increasing self-efficacy and bolstering engagement [30].

We also observed two engagement personas that demonstrated a strong affinity for specific features of the digital DPP: Data-Driven Members regularly weighed in, and Learners had high engagement with educational content. Weight loss is the primary goal of the DPP, and Data-Driven Members seemed particularly motivated by tracking the data most related to this goal. Self-weighing has a well-established relationship with weight loss [31], and a greater frequency of weigh-ins has been associated with other healthy behaviors including less sedentary time, more physical activity, and healthier eating [32]. In contrast, Learners were health-information-seeking members who seemed to prioritize learning the educational material provided by the DPP. Education plays an important role in self-management for people living with prediabetes. Attending the educational lessons of the DPP is important for clinical outcomes, with each additional session attended associated with 0.3% additional weight loss [33]. The distinctive engagement patterns of Data-Driven Members and Learners highlight ways to further personalize the delivery of the app experience for each engagement persona.

People who enjoy tracking their data tend to be motivated by seeing their progress expressed numerically and comparing their results to others’ [34]. Gamification, such as a point system or rewards for engagement in behaviors that support weight loss, may be one way to further personalize the member experience for Data-Driven Members [35]. For example, Data-Driven members could be offered points based on achievements such as earning badges for completing educational lessons. The accumulation of badges could lead to earning a reward incentive such as a Fitbit, and members would be able to track their progress toward earning the reward. In contrast, Learners may benefit from strategies to help translate educational content into behavior change, since the educational check-ins were their primary mode of engagement. Knowledge alone is not necessarily enough to change behavior, and emphasizing problem-solving, self-empowerment, and motivation(s) for change may be an important focus for Learners [36]. Learners may additionally benefit from targeted educational content or coaching exchanges that emphasize the benefits of self-weighing since this was the one app behavior that distinguished them from Enthusiasts. These types of delivery personalization would enable each member to use the digital DPP in the ways that they find most enjoyable, with the artificial intelligence coach ensuring that they still benefit from all aspects of the program.

The digital DPP examined in this study is already a personalized program powered by artificial intelligence. The coach leverages a member’s unique data (e.g., meals logged, physical activity, sleep) to offer personalized insights and suggestions. However, much like a human coach, it takes time for the artificial intelligence coach to learn how a member prefers to be coached and in what areas they might require additional support. Rather than altering the content that a member receives, the discovery of distinctive engagement patterns can help the artificial intelligence coach to tailor the delivery of this personalized content, coaching, and feedback to be most palpable and beneficial to each member. Identifying distinctive patterns of engagement also enables the coach to determine if a member’s actions match their stated goals. The artificial intelligence coach can facilitate greater awareness of self through identifying mismatches between goals and behaviors [37]. Engagement personas may be used by the coach to predict pitfalls and help overcome obstacles to success during a time when a member is engaged enough to readjust their behaviors and trajectory. Thus, identifying an engagement persona may be used to guide new strategies for re-engaging members who are beginning to disengage.

### 4.2. Future Directions

Bivariate clustering enabled us to describe engagement patterns that would not have been clear had we limited our investigations to only univariate behaviors. For example, we observed that certain app behaviors, such as frequent weigh-ins, helped to distinguish groups of low and high program engagers. Thus, frequent weighing may be an important behavior indicative of program engagement. These types of insight facilitate the tailoring of coaching for specific patterns of engagement, as described above. For this study, we based the program features on the required National DPP curriculum, so the goal would be to optimize app features in the delivery of the DPP. However, the discovery of engagement personas is beneficial not only to inform future enhancements of a digital DPP but more broadly to inform a variety of digital health interventions. Digital health platforms are increasing in popularity for the management of a variety of chronic health conditions [2]. Determining how app use and educational content interact can provide insight into the “active ingredients” associated with program success.

Digital technologies are dynamic entities that are continuously refined and updated. There will always be a certain degree of heterogeneity in the program experience across different member cohorts. We plan to apply this method for discovering engagement personas to a much larger population to obtain a more stable sample average of longitudinal engagement patterns. We also have additional questions about the engagement personas discovered in this study. For example, does engagement persona membership at 90 days persist to the conclusion of the year-long program? Is it possible to identify an engagement persona earlier than 90 days? How malleable is an engagement persona? Can we use targeted engagement strategies to help a Casual Member or Mainstream Member to change engagement personas and increase their likelihood of retention? To address these questions, we plan to investigate engagement persona membership at different time points during the year-long program.

We also plan to explore the engagement personas in the context of more detailed clinical outcomes to better elucidate the relationship between engagement in a digital DPP and clinical outcomes, which is a nascent area of research. While our preliminary investigation of weight loss suggests that there are differences among the engagement personas, would this remain true at one year? A member may get what they need from a program with very different engagement patterns; there may be more than one path to success. Indeed, members of all engagement personas achieved some weight loss over the first four months of the digital DPP. Thus, a critical next step is to recognize when and where it may be appropriate to make modifications to a member’s experience based on the information gained from their engagement persona membership.

Indirect clinical outcomes, such as self-reported medication adherence, are also of interest. Recent evidence suggests that some individuals with prediabetes benefit from taking medications, such as Metformin, in addition to participating in the DPP [38]. Medication adherence is often suboptimal, and strategies to promote compliance are necessary [39]. Medication adherence is one of several recent features added to the digital DPP investigated in this study. Future exploration of the engagement personas should also include outcomes-related features such as medication adherence.

A complementary area for future exploration is identifying barriers to engagement specific to each engagement persona, along with corresponding interventions (facilitators) that effectively address these barriers. Specific barriers may include health beliefs, low intrinsic motivation, skills deficits, low insight or awareness, reactance, or other psychological factors that may affect engagement and outcomes [40]. Pinpointing barriers that accompany each engagement persona could allow for targeted, multicomponent interventions that facilitate engagement and adherence to digital behavioral health programs.

### 4.3. Strengths and Limitations

We employed an unsupervised learning approach for discovering engagement personas that imposed no a priori expectation on the number of engagement patterns and is flexible enough to be applied to other digital health programs. We used real member data from a commercially available, CDC-recognized DPP, and the collection of these data imposed no burden on members (i.e., did not interfere with their natural behavior). We included members with a wide range of engagement patterns from Casual Members to Enthusiasts and investigated preliminary differences in the clinical outcomes associated with these distinctive engagement patterns.

We did not identify an engagement persona for all members in this study and, further, did not observe complete persistence of members in each engagement persona during statistical validation. However, these differences do not reduce the utility of the univariate and bivariate clustering techniques. Engagement personas take time to refine, and we expect improvements in both the percentage of members with an identified engagement persona and the persistence of the engagement personas as the sample size for model training increases. The clinical validation of the engagement personas should be considered exploratory since we did not have complete data for some member characteristics and clinical outcomes. Finally, just as there are limitations to qualitative techniques for developing engagement personas, there are also inherent limitations to purely quantitative techniques. Behavioral patterns represent an important aspect of engagement but do not capture other aspects such as affective and cognitive investment in a program [41]. We plan to explore these engagement personas further by comparing them to members’ motivations and goals, which could be solicited through qualitative interviews conducted by the artificial intelligence coach or survey feedback.

## 5. Conclusions

Digital health technologies are shaping the future of preventive health care. To realize their full potential, we must identify the wants, needs, and preferences of members and the barriers to success. This study presented a method for discovering engagement personas based on distinctive, longitudinal engagement patterns in a digital DPP. The resulting engagement personas differed in member demographics, characteristics, and clinical outcomes. Enthusiasts represented the oldest members who engaged in all app- and program-specific features, and Enthusiasts achieved the greatest weight loss of 4.5% four months into the program. Other personas represented members who differentially favored engaging with specific app or program features, such as weigh-ins for Data-Driven members and educational lessons for Learners. We further identified engagement personas of members requiring additional support to increase their engagement: Casual and Mainstream Members. The engagement personas provide actionable insight into how to tailor the delivery of program content, coaching, and feedback for members with specific patterns of observed engagement. The method proposed for discovering engagement personas in this study will facilitate further tailoring of the digital program experience and, ultimately, ensure that individuals who choose to participate in a digital DPP achieve maximal clinical benefits.

## Figures and Tables

**Table 1:** Contingency table showing counts per each label pair resulting from the five app-feature and four program-feature clusters. Engagement personas identified by cross-referencing cells containing a high proportion (≥60%) of marginal total members (each persona shown in dark shading).

		Program-Feature Cluster Label				Total
		1	2	3	4	
App-Feature Cluster Label	1	79	24	12	242	357
	2	303	14	1	4	322
	3	7	74	306	42	429
	4	222	17	2	27	268
	5	4	33	146	54	237
Total		615	162	467	369	1613

**Table 2:** Comparisons of engagement personas on participant demographics, characteristics, weight loss, and engagement metrics across 90 days.

	Persona Not Identified ^1^ (*n* = 394)	Casual Members ^2^ (*n* = 303)	Mainstream Members ^3^ (*n* = 242)	Learners ^4^ (*n* = 306)	Data-Driven Members ^5^ (*n* = 222)	Enthusiasts ^6^ (*n* = 146)	All (*n* = 1613)
	Mean (SE) % of *n* if <100						
Age (years)	50.9 (0.5) ^2,4,5^	46.3 (0.6) ^1,3,4,6^	49.8 (0.7) ^2,4,6^	53.2 (0.5) ^1,2,3,5^	47.4 (0.6) ^1,4,6^	53.5 (0.8) ^1,2,3,5^	50.1 (0.3)
Body mass index (kg/m^2^)	37.3 (0.4) ^2^ 82%	39.5 (0.5) ^1,3,4,5,6^ 84%	37.4 (0.5) ^2^ 95%	37.2 (0.4) ^2^ 95%	37.5 (0.4) ^2^ 93%	35.7 (0.6) ^2^ 97%	37.5 (0.2) 90%
% weight loss at 4 months	2.7 (0.2) ^6^ 65%	1.6 (0.2) ^5,6^ 29%	2.0 (0.2) ^6^ 52%	2.6 (0.2) ^6^ 82%	3.1 (0.3) ^2,6^ 80%	4.5 (0.3) ^1,2,3,4,5^ 95%	2.8 (0.1) 64%
# of weigh-ins	29.0 (1.4) ^2,3,4,5,6^	6.4 (0.5) ^1,3,4,5,6^	16.8 (0.9) ^1,2,5,6^	19.7 (0.7) ^1,2,5,6^	39.4 (2.1) ^1,2,3,4,6^	70.9 (2.9) ^1,2,3,4,5^	26.4 (0.7)
# of meals logged	96.2 (3.5) ^2,4,5,6^	16.1 (0.8) ^1,3,4,6^	87.8 (3.0) ^2,4,5,6^	189.8 (4.8) ^1,2,3,5^	26.6 (1.2) ^1,3,4,6^	202.7 (8.9) ^1,2,3,5^	97.7 (2.3)
# of coaching exchanges	115.6 (3.0) ^2,4,5,6^	31.9 (1.0) ^1,3,4,6^	114.7 (3.3) ^2,4,5,6^	219.0 (5.4) ^1,2,3,5,6^	47.9 (1.5) ^1,3,4,6^	246.7 (12.5) ^1,2,3,4,5^	121.9 (2.6)
# of check-ins	34.9 (0.8) ^2,4,5,6^	6.7 (0.3) ^1,3,4,5,6^	35.7 (0.6) ^2,4,5,6^	71.2 (0.7) ^1,2,3,5^	11.6 (0.4) ^1,2,3,4,6^	68.9 (1.1) ^1,2,3,5^	36.5 (0.7)
	% *n* % of *n* if <100						
Gender (% female)	70% ^2^	60% ^1,3^	76% ^2,4,6^	61% ^3^	67%	63% ^3^	66%
Race (% white)	72% 96%	68% 96%	74% 95%	72% 92%	70% 97%	77% 94%	72% 95%
Ethnicity (% Hispanic or Latino)	10% 96%	12% 96%	10% 95%	13% 92%	8% 97%	9% 94%	10% 95%
% in Facebook Group	46% ^2,5,6^	20% ^1,3,4,5,6^	42% ^2,5,6^	44% ^2,5,6^	29% ^1,2,3,4,6^	65% ^1,2,3,4,5^	39%

Note: Each engagement persona is labeled with a superscript 1–6. Superscripts within each cell indicate Tukey pairwise significant differences between personas at *p* < 0.05.

## Data Availability

The data presented in this study are available from the corresponding author on reasonable request. The data are not publicly available because they are the intellectual property of a commercial product.

## Appendix A

Statistical validation of the engagement personas using (1) An alternative, hierarchical clustering approach on the initial training set of members and (2) The same k-means approach on an independent test set.

### Appendix A.1. Results of the Statistical Validation: Hierarchical Clustering

Using the original training set of N=1613 members, we performed unsupervised clustering with a different statistical approach: hierarchical clustering with Ward’s method. We discovered seven univariate app-feature clusters (compared to five with k-means) and four program-feature clusters (the same as with k-means). Thus, the optimal number of univariate clusters discovered with hierarchical clustering may differ from the optimal number with k-means. Using the original cross-referencing threshold of 60% during bivariate clustering, we observed the same five personas (for the persistence in each persona see Table A1). The hierarchical approach identified 69% of members as belonging to a persona (compared to 76% with k-means). We also observed a sixth engagement persona that represented a split of the Mainstream Members into two personas with similar patterns of engagement but one above the sample average and the other below. As a reminder, the general shape of Mainstream Members in the initial training set was that of initially higher app and program engagement than the sample average but whose engagement with both features dropped substantially over 90 days.

**Table A1:** The percent of members persisting for each persona when using the alternative hierarchical clustering method.

Persona	% of Members Persisting
Not Identified	71%
Casual Members	38%
Mainstream Members	69%
Learners	82%
Data-Driven	57%
Enthusiasts	79%

### Appendix A.2. Results of the Statistical Validation: Independent Test Set

Using an independent test set of N=1071 DPP members, we validated the initial k-means clustering approach. We obtained personas for 72% of the sample. We observed six univariate app-feature clusters and four program-feature clusters. However, to observe the original five personas during bivariate clustering, we had to lower the cross-referencing threshold to 50%. This was not a large change since there was no difference between the 50% and 60% thresholds during this step with the original cohort. In addition to the five original personas, we discovered a sixth persona that looked similar to Mainstream Members but with a steeper rate of drop off in engagement over 90 days. The breakdown of members among the personas was 18% Casual Members, 19% Mainstream Members (variation 1), 9% Mainstream Members (variation 2), 17% Learners, 7% Data-Driven Members, and 30% Enthusiasts. The observed differences between cohorts may have been due to differences in the sample average time series. For example, the test cohort of members was more highly engaged with both app and program features than the training cohort. On average, the test cohort had a greater number of days with coaching exchanges (49 vs. 42; *p* ≤ 0.0001), meals logged (44 vs. 35; *p* ≤ 0.0001), weigh-ins (20 vs. 18; *p* ≤ 0.0001), and check-ins with program content (38 vs. 32; *p* ≤ 0.0001) over 90 days. Increasing the sample size for training in the future would likely help to stabilize the sample averages in the univariate clusters.
